# Transmission of Grapevine Red Blotch Virus by *Spissistilus festinus* [Say, 1830] (Hemiptera: Membracidae) between Free-Living Vines and *Vitis vinifera* ‘Cabernet Franc’

**DOI:** 10.3390/v14061156

**Published:** 2022-05-26

**Authors:** Victoria Hoyle, Madison T. Flasco, Jiyeong Choi, Elizabeth J. Cieniewicz, Heather McLane, Keith Perry, Gerald Dangl, Maher Al Rwahnih, Michelle Heck, Greg Loeb, Marc F. Fuchs

**Affiliations:** 1 School of Integrative Plant Science, Plant Pathology and Plant-Microbe Biology, Cornell University, Geneva, NY 14456, USA; mf725@cornell.edu (M.T.F.); jc3398@cornell.edu (J.C.); hlm9@cornell.edu (H.M.); marc.fuchs@cornell.edu (M.F.F.); 2 Plant and Environmental Sciences, Clemson University, Clemson, SC 29634, USA; ecienie@clemson.edu; 3 School of Integrative Plant Science, Plant Pathology and Plant-Microbe Biology, Cornell University, Ithaca, NY 14853, USA; klp3@cornell.edu (K.P.); mlc68@cornell.edu (M.H.); 4 Department of Plant Pathology, Foundation Plant Services, University of California, Davis, CA 95616, USA; gsdangl@ucdavis.edu (G.D.); malrwahnih@ucdavis.edu (M.A.R.); 5 Emerging Pests and Pathogens Research Unit, USDA Agricultural Research Service, Robert W. Holley Center for Agriculture and Health, Ithaca, NY 14853, USA; 6 Department of Entomology, Cornell University, Geneva, NY 14456, USA; gme1@cornell.edu

**Keywords:** *Vitis californica*, *Vitis vinifera*, *Geminiviridae*, *Grablovirus*, *Spissistilus festinus*

## Abstract

Grapevine red blotch disease emerged within the past decade, disrupting North American vine stock production and vineyard profitability. Our understanding of how grapevine red blotch virus (GRBV), the causal agent of the disease, interacts with its *Vitis* hosts and insect vector, *Spissistilus festinus*, is limited. Here, we studied the capabilities of *S. festinus* to transmit GRBV from and to free-living vines, identified as first-generation hybrids of *V. californica* and *V. vinifera* ‘Sauvignon blanc’ (Vcal hybrids), and to and from *V. vinifera* ‘Cabernet franc’ (Vvin Cf) vines. The transmission rate of GRBV was high from infected Vcal hybrid vines to healthy Vcal hybrid vines (77%, 10 of 13) and from infected Vvin Cf vines to healthy Vcal hybrid vines (100%, 3 of 3). In contrast, the transmission rate of GRBV was low from infected Vcal hybrid vines to healthy Vvin Cf vines (15%, 2 of 13), and from infected Vvin Cf vines to healthy Vvin Cf vines (19%, 5 of 27). No association was found between transmission rates and GRBV titer in donor vines used in transmission assays, but the virus titer was higher in the recipient leaves of Vcal hybrid vines compared with recipient leaves of Vvin Cf vines. The transmission of GRBV from infected Vcal hybrid vines was also determined to be trans-stadial. Altogether, our findings revealed that free-living vines can be a source for the GRBV inoculum that is transmissible by *S. festinus* to other free-living vines and a wine grape cultivar, illustrating the interconnected roles of the two virus hosts in riparian areas and commercial vineyards, respectively, for virus spread. These new insights into red blotch disease epidemiology will inform the implementation of disease management strategies.

## 1. Introduction

Grapevine red blotch virus (GRBV) is an emerging pathogen and the causal agent of grapevine red blotch disease [1]. This virus has been a concern of the North American grape and wine industries for the past decade [2,3,4,5]. Infected *Vitis vinifera* (Vvin) vines express foliar symptoms, such as reddening or chlorosis on black- and white-berried cultivars, respectively, and can produce poorly ripened berries with reduced sugar and anthocyanin accumulation and lower bunch weights [5,6,7]. The presence of GRBV can negatively affect vine productivity, wine quality and sensory attributes, and vineyard profitability. The economic impact of GRBV is estimated to range between $2213 and $68,548 per hectare over the lifespan of a diseased vineyard [8].

GRBV is a member of the species *grapevine red blotch virus* of the genus *Grablovirus* in the family *Geminiviridae* [3,5]. The DNA genome of GRBV is single-stranded and circular with seven bidirectional open reading frames (ORFs). Four ORFs are in the viral sense orientation and encode the coat protein (V1) and viral movement proteins (V2 and V3). The function of V0 is unknown [9,10,11,12]. The remaining three ORFs are in the complementary sense orientation and encode a replication-associated protein (C1 and C2) and a protein (C3) for which the function is not yet determined [9,10,12].

The widespread distribution of GRBV has been recorded in vineyards throughout the United States [4,9,13,14,15] and Canada [16,17,18]. GRBV has also been reported in Switzerland [19], South Korea [20], Mexico [21], Argentina [22], India [23], Italy [24], and France [25]. The global reach of GRBV can be attributed to the dissemination of infected planting materials, while secondary spread in U.S. states, such as California [3,26,27] and Oregon [28,29], as well as the province of British Columbia in Canada [16], is ascribed to an insect vector. There is no evidence of secondary spread in New York [27], Switzerland [19], or France [25].

In vineyards where secondary spread is documented, spatiotemporal increase patterns of GRBV-infected vines point to the presence of an aerial hemipteran vector [3,16,26,27,28,29]. Of the few vector candidates identified in a diseased ‘Cabernet franc’ vineyard in Napa County in northern California, *Spissistilus festinus* [Say, 1830] (Hemiptera: Membracidae), the three-cornered alfalfa hopper, proved to be the most likely candidate [3] and was subsequently reported to vector GRBV [30,31]. Other hemipteran vector candidates were recently described in California [32,33].

The transmission of GRBV by *S. festinus* is circulative and non-propagative, i.e., GRBV can only be transmitted if the virus transits through the salivary glands of *S. festinus* following acquisition, but the virus does not replicate in the insect vector [31]. In comparison with other members of the family *Geminiviridae*, *S. festinus*-mediated transmission of GRBV has been demonstrated to be comparatively inefficient, with an extended acquisition period (AAP, 10 days) and an extended inoculation access period (IAP, 4 days) on Vvin ‘Cabernet franc’ (Cf) grapevines [31]. These atypical transmission characteristics reveal a unique relationship between the *S. festinus*, GRBV, and *Vitis* species and highlight the need to further our understanding of transmission biology and disease epidemiology.

In addition to Vvin, rootstock genotypes [2,3], interspecific hybrids [34], and Muscadine grapes [15], GRBV has been found in free-living vines in northern California [26,31,35,36] and southern Oregon [29]. In contrast, the virus was not detected in free-living vines in New York [26]. These findings suggested that free-living vines could serve as GRBV inoculum sources in northern California and southern Oregon, although, unlike for Vvin cultivars, free-living vines sustain latent infections [26,29]. Given that one of the previous surveys of free-living vines in northern California targeted plants that were at least 100 m apart [26], an aerial hemipteran vector is likely involved in GRBV spread within populations of free-living vines. We hypothesized that *S. festinus* can transmit GRBV between free-living and wine grape cultivars. Here, we summarize the results of a study designed to provide new insights into red blotch disease epidemiology by determining whether free-living vines play an active role as GRBV reservoirs.

## 2. Materials and Methods

### 2.1. Plant Materials

Hardwood cuttings of healthy and GRBV-infected Vvin Cf vines were collected in New York vineyards during the winters of 2015–2018. Cuttings were grown to maturity in the greenhouse with conditions set to 22 ± 3 °C and a 16 h:8 h light:dark photoperiod. Green cuttings were also propagated from actively growing shoots of healthy Vvin Cf in the summer of 2020. ‘Cabernet franc’ was selected in this study as a representative cultivar of wine grape production vineyards.

Hardwood cuttings of healthy and GRBV-infected free-living vines were collected from Napa County in California in 2017–2018. These free-living vines were most likely *Vitis californica* (Vcal) or Vcal hybrids based on previous genotyping work [26]. To determine ancestry of these vines, genotyping at eight simple-sequence repeat (SSR) loci previously shown to distinguish Vcal from Vvin and uniquely identify Vvin cultivars was performed [35,36]. At each locus, alleles were ascribed to Vcal or other *Vitis* species. To determine possible parent cultivars, alleles not ascribed to Vcal were compared to reference profiles of scion and rootstock cultivars currently and historically grown in northern California [35,36].

GRBV-infected free-living vines determined to be Vcal hybrids and GRBV-infected Vvin Cf vines were used as donor plants in transmission assays with *S. festinus*, as previously described [31]. Excised leaves of Vvin Cf vines and Vcal hybrid vines that tested negative for GRBV by diagnostic PCR [9,31] were used as recipient materials of GRBV in *S. festinus*-mediated transmission assays [31]. Healthy and infected materials from the two *Vitis* species were used as controls in diagnostic PCR for GRBV.

*Phaseolus vulgaris* ‘Hystyle’ was used as a rearing host of *S. festinus* and as recipient plant material in trans-stadial transmission assays. Snap bean plants were set and maintained in greenhouses and later moved to controlled environmental chambers with the following growing conditions: 25 °C, 16 h:8 h light:dark photoperiod, and 80% relative humidity.

### 2.2. Transmission Assays of GRBV by S. festinus

**Figure 1:**
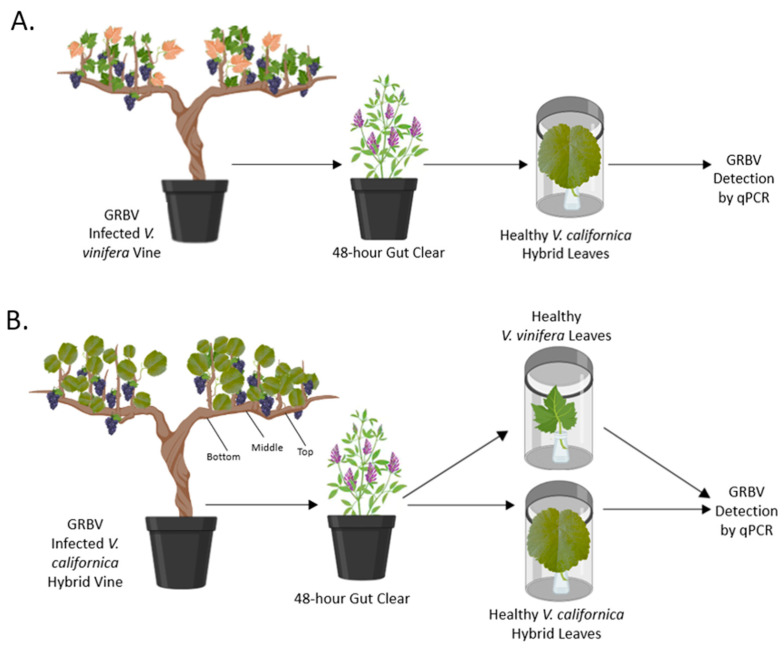
Schematic representation of the three transmission assays performed in this study. (**A**) Transmission assay used GRBV-infected *Vitis vinifera* ‘Cabernet franc’ as donor material and healthy detached *V. californica* hybrid leaves as the recipient. (**B**) Transmission assays used GRBV-infected *V. californica* hybrids as donor material and healthy, detached leaves from either *V. vinifera* or *V. californica* hybrid as recipient material. Virus donor plants were grown from hardwood cuttings of GRBV-infected cultivated or free-living vines collected from northern California. Healthy excised leaves were placed in parafilm-covered 2-mL collection vials filled with sterile water and inserted into sealed polypropylene containers for exposure to *Spissistilus festinus*. The artwork was produced using the program BioRender (Toronto, ON, Canada).

Three transmission experiments were conducted to determine the attributes of GRBV spread by *S. festinus* from and to free-living Vcal hybrids. Transmission assays were conducted using insects collected from alfalfa fields in northern California, then reared on healthy ‘Hystyle’ snap beans in environmentally controlled chambers. Assays utilized either Vcal hybrid vines or Vvin Cf vines as donor and recipient plant material (Figure 1). In transmission assays where Vvin Cf vines were used as the virus donor plant and leaves of healthy Vcal hybrid vines were the recipient (Figure 1A), *S. festinus* were allowed to feed for 30 days on GRBV-infected vines and then had a gut clearing period for 48-h on alfalfa, a non-host of GRBV [31]. Next, cohorts of five *S. festinus* were moved to each healthy detached leaf from Vcal hybrid vines using an aspirator (Gemplers RHM200 D-Cell Powered Aspirator) to feed for four days, as previously described [31]. Finally, insects were removed using an aspirator, and the detached Vcal hybrid leaves were tested for GRBV by PCR [9] after 10–14 additional days. This was repeated in four chambers, three with insects from GRBV-infected Vvin vines and one as the healthy control using non-viruliferous, colony-reared *S. festinus*.

Additional transmission assays relied on Vcal hybrid vines as the donor plants and used excised leaves of Vcal hybrid vines or Vvin Cf vines as the recipient plant tissue (Figure 1B). In the initial transmission assay, *S. festinus* were confined exclusively to the top, middle, or bottom of shoots of GRBV-infected Vcal hybrid vines using insect rearing sleeves (BugDorm Insect Rearing Sleeve, L70 × W30 cm) to feed for two or three weeks in the AAP, respectively, and then placed on alfalfa for a 48-h gut clearing period. Next, cohorts of five *S. festinus* were randomly selected and placed on excised leaves of either Vcal hybrid vines or Vvin Cf vines to feed for five days (Figure 1B). Insects and plant material were collected and tested as previously described for the assay using Vvin Cf vines as donor plant material.

The base of the petiole of each excised leaf to be used as recipient materials in transmission assays was removed using a sterile blade and tested for GRBV by PCR at the onset of the experiments [9,31]. After exposure to *S. festinus*, petiole tissue and leaf tissue close to the midrib were tested separately for GRBV by PCR [9,31]. Virus test results obtained with petiole and leaf tissue at the completion of transmission assays were combined.

### 2.3. Trans-Stadial Transmission of GRBV by S. festinus

**Figure 2:**
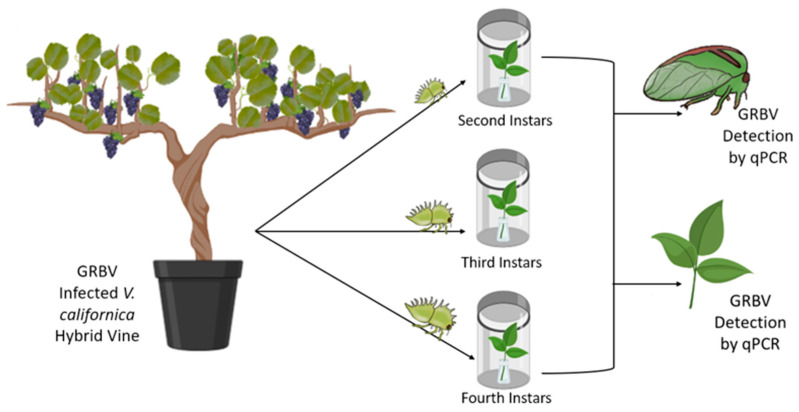
Schematic representation of the trans-stadial transmission assays performed in this study. Assays used GRBV-infected *Vitis californica* hybrid vines as donor material and healthy, detached leaves from *Phaseolus vulgaris* as recipient material. Virus donor plants were grown from hardwood cuttings of GRBV-infected free-living vines collected from northern California. Healthy excised snap bean leaves were placed in parafilm-covered 2-mL collection vials filled with sterile water and inserted into sealed polypropylene containers for exposure to second, third, or fourth instar *Spissistilus festinus* until adulthood. The artwork was produced using adapted art made by Brandon Roy and the program BioRender (Toronto, ON, Canada).

To test for trans-stadial transmission of GRBV, non-viruliferous adult *S. festinus* were exposed to caged GRBV-infected Vcal hybrid vines until nymphs emerged. Then, the second, third and fourth nymphal stages were separately transferred with a fine-tipped paintbrush onto detached, healthy snap bean trifoliates in clip cage chambers. As soon as nymphs molted into adulthood, adult insects were tested for GRBV by qPCR [31,36]. Additionally, petioles of snap bean trifoliates were sampled at the onset of the assays and again following the removal of adults and were tested for GRBV by qPCR to assess virus transmission (Figure 2).

### 2.4. Assessing the Distribution of GRBV in Free-Living Vines

The distribution of GRBV in free-living vines was assessed in leaves of three Vcal hybrid vines growing in a greenhouse, two derived from vines in Napa County that previously tested positive for GRBV, and one derived from a vine in Napa County that previously tested negative for GRBV. Single leaf samples were collected from two shoots per vine with three samples taken from each of the top, middle, or bottoms of the shoots for a total of six leaves per shoot location and eighteen leaves per vine. The petiole of individual leaves was tested for the presence of GRBV by qPCR [31,37].

### 2.5. Nucleic Acid Extraction from Plant Tissue and S. festinus, and GRBV Detection by PCR and qPCR

Genomic DNA was isolated from plant tissue using the H.P. Plant DNA Mini kit (OMEGA Bio-tek, Norcross, GA, USA) or the MagMAX™-96 Al/ND Isolation Kit (ThermoFisher Scientific, Waltham, MA, USA) on a KingFisher™ instrument. Genomic DNA was isolated from *S. festinus* using the MagMAX™ DNA Multi-Sample Ultra Kit (ThermoFisher Scientific, Waltham, MA, USA) on a KingFisher™ instrument. Nucleic acid preparations from plant material and *S. festinus* were used in diagnostic GRBV multiplex PCR [9,31] and quantification of GRBV by qPCR [31,37].

The presence of GRBV in plant tissue and *S. festinus* was tested by multiplex PCR using genomic DNA and primer pairs designed in the ORF coding for the coat protein (CP) and replication-associated proteins (RepA), as previously described [31]. DNA amplicons were analyzed by agarose gel electrophoresis and visualized under UV illumination after staining with GelRed^®^ (Biotium, Fremont, CA, USA).

GRBV was also tested in nucleic acids from plant tissue by qPCR with SYBR^®^ Green reagents (iTaq Bio-Rad Universal SYBR^®^ Green Supermix, Hercules, CA, USA). For the detection of GRBV in DNA isolated from *Vitis* samples, primers pREP3v and pREP4v designed in GRBV ORF C1 coding RepA and a primer pair designed in the plant nicotinamide adenine dinucleotide phosphate (NADP) gene were used [37]. For the testing of GRBV in snap bean trifoliates, the same GRBV primers pREP3v and pREP4v were used in addition to a primer pair designed in the bean housekeeping gene *T197* coding a guanine nucleotide-binding protein beta subunit-like protein [38]. Negative controls included sterile water and nucleic acids from healthy plants.

GRBV testing in *S. festinus* by qPCR required primers pREP3v and pREP4v [37], *S. festinus* primers Sf18SFor and Sf18SRev [4], SYBR^®^ Green reagents, and 50 ng of genomic DNA from individual specimens. Negative controls included sterile water and nucleic acids isolated from *S. festinus* from the colony maintained on *P. vulgaris*.

For virus quantification in plant samples and insect specimens by qPCR, each reaction was conducted in triplicate (three technical replicates) on a Biorad C1000 Touch thermocycler. GRBV was quantified using the relative ∆∆Ct method to calculate the fold difference of GRBV DNA between two samples.

### 2.6. Statistical Analyses

Statistical analyses were performed on the titers of GRBV in plant tissue and insect specimens, according to qPCR Ct values with ANOVA in R Studio. Fold differences in GRBV titer were based on ΔΔCt values. Statistically significant differences between ΔΔCt expression fold change values (2^(−ΔΔCt)^) in excised leaves and adult *S. festinus* were determined using Tukey’s Honestly Significant Difference (HSD) test. The significance level was set at α = 0.05.

## 3. Results

### 3.1. Identification of the Free-Living Vines Used in This Study and GRBV Distribution

The dormant cuttings of free-living vines from Napa County in California that previously tested negative or positive for GRBV by PCR [26,35] were grown in the greenhouse. No phenotypic differences between healthy and GRBV-infected vines were observed, in agreement with previous observations of latent infections in free-living vines [26,29]. Mature leaves were collected and genotyped using eight specific SSR markers. Genotyping profiles revealed that the free-living vines selected for this study corresponded to first-generation hybrids of Vcal and Vvin ‘Sauvignon blanc’, regardless of their infectious status.

**Figure 3:**
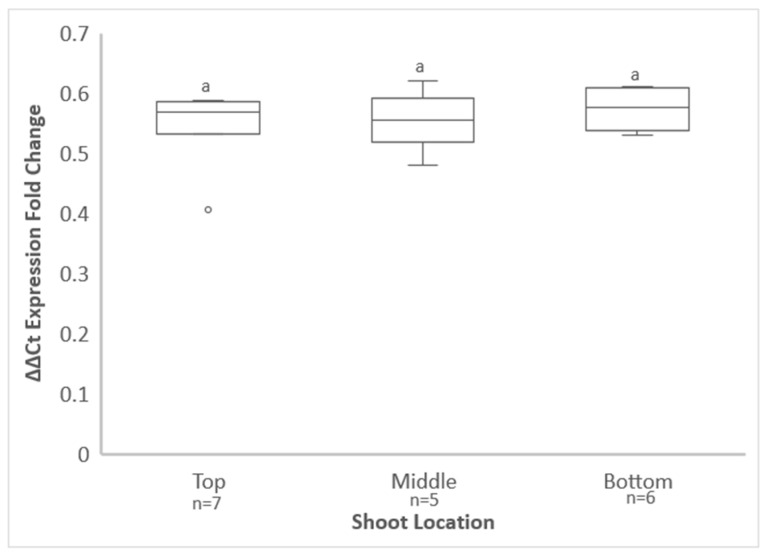
Quantification of grapevine red blotch virus in leaves of the top, middle, and bottom sections of shoots of *Vitis californica* hybrid vines by qPCR. Data show the ΔΔCt expression fold change values (2^(−ΔΔCt)^) from five to seven leaf samples of two vines for each shoot section. Vertical axes are set on logarithmic scales. Error bars indicate standard error of the mean. Lowercase lettering indicating insignificant differences between 2^(−ΔΔCt)^ values, as determined by Tukey’s honestly significant difference test (*p* < 0.05), are shown above standard error bars.

GRBV was detected throughout the two infected Vcal hybrid vines with an average of half of the leaf samples testing positive for each of the three shoot locations, as shown by qPCR, although slight differences in percentage of positive leaves by location were noticed between the two vines (Table 1). No statistically significant difference in GRBV titer was found based on the location of leaf collection (*p* = 0.5291) (Figure 3). As expected, the virus was not detected in leaf tissue from the free-living vine that initially tested negative for GRBV.

### 3.2. GRBV Transmission by S. festinus from Infected Free-Living Vines

Two independent *S. festinus*-mediated GRBV transmission experiments were performed from infected Vcal hybrid vines to detached leaves of healthy Vcal hybrid vines. For the first experiment, given the presence of GRBV throughout the vines, despite an uneven distribution, *S. festinus* were caged directly on the vines’ top, middle, or lower canopies using insect sleeves with an AAP of 14 days. Transmission of GRBV to healthy, detached leaves of Vcal hybrid vines occurred when *S. festinus* fed on tissue from the middle (100%, 3 of 3) and bottom (50%, 1 of 2) shoots, as shown by multiplex PCR. Transmission also occurred from infected Vcal hybrid vines to healthy detached leaves of Vvin Cf when *S. festinus* fed on tissue from the middle (66%, 2 of 3) but not the bottom (0%, 0 of 2) shoots. Similarly, transmission did not occur from infected Vcal hybrid vines to healthy, detached leaves of either Vcal hybrid vines (0%, 0 of 2) or Vvin Cf vines (0%, 0 of 2) when *S. festinus* were confined on the top of the shoots.

For the second transmission experiment, we restricted the *S. festinus* to the middle of shoots and allowed for an extended AAP of one additional week (three weeks total). In replicated experiments, these conditions resulted in a high transmission rate (100%, 6 of 6) from GRBV-infected Vcal hybrid vines to healthy, detached leaves of Vcal hybrid vines.

By combining data from the two transmission experiments, the rate of *S. festinus*-mediated GRBV transmission from infected Vcal hybrid vines to healthy, detached leaves of Vcal hybrid vines was high (77%, 10 of 13) (Table 2). In contrast, a low transmission rate (15%, 2 of 13) was obtained in replicated assays from GRBV-infected Vcal hybrid vines to healthy, detached leaves of Vvin Cf (Table 2). These results documented the ability of *S. festinus* to transmit GRBV from infected free-living to healthy free-living vines and from infected free-living to healthy cultivated vines.

### 3.3. GRBV Transmission by S. festinus from Infected ‘Cabernet Franc’

Transmission assays from infected Vvin Cf vines to healthy Vcal hybrid vines revealed GRBV was transmitted to all detached leaves (100%, 3 of 3), as shown by multiplex PCR (Table 2). No transmission occurred from healthy Vvin Cf grapevines to healthy, detached leaves of Vcal hybrid vines (0%, 0 of 3), as expected. These results were consistent with *S. festinus*-mediated transmission of GRBV from an infected wine grape cultivar, i.e., Vvin Cf, to free-living vines, i.e., Vcal hybrids. In addition, replicated assays resulted in a low transmission rate of GRBV from infected Vvin Cf vines to detached leaves of healthy Vvin Cf vines (19%, 5 of 27) (Table 2), confirming previous findings [31].

### 3.4. Comparative GRBV Titer in Infected Free-Living and ‘Cabernet Franc’ Vines

**Figure 4:**
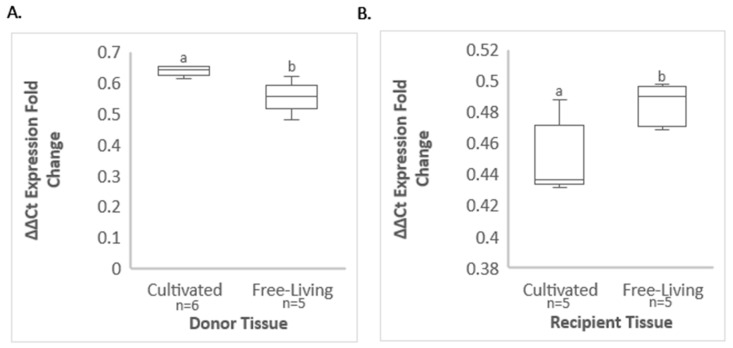
Quantification of grapevine red blotch virus (GRBV) by qPCR in (**A**) donor *Vitis californica* hybrid vines (Free-living) in comparison with donor *V. vinifera* ‘Cabernet franc’ vines (Cultivated), and (**B**) recipient detached *V. californica* hybrid leaves exposed to *Spissistilus festinus*, which fed on GRBV-infected *V. californica* hybrid vines, in comparison to *V. vinifera* ‘Cabernet franc’ leaves exposed to *S. festinus*, which fed on GRBV-infected *V. vinifera* vines. Error bars indicate standard error of the mean. Lowercase lettering indicates significant differences between ΔΔCt expression fold change values (2^(−ΔΔCt)^) values, as determined by Tukey’s honestly significant difference test (*p* < 0.05) and are shown above standard error bars.

The virus titer in donor and recipient materials used in transmission assays was determined by qPCR (Figure 4). A significantly higher GRBV titer was observed in infected Vvin Cf vines (2^(−ΔΔCt)^ = 0.639) compared with infected Vcal hybrid vines (2^(−ΔΔCt)^ = 0.555) (*p* = 0.003) used as donor plants in transmission assays (Figure 4A). Additionally, a significantly higher GRBV titer was obtained in the excised leaves of Vcal hybrid vines inoculated by insect vectors (2^(−ΔΔCt)^ = 0.485) compared with the excised leaves of Vvin Cf vines used as recipients of insect vector-mediated transmission (2^(−ΔΔCt)^ = 0.449) (*p* = 0.0174) (Figure 4B). These results revealed an inverse correlation between virus titer in donor vines and transmission efficiency and a positive association between transmission efficiency and virus titer in the excised leaves used as recipients.

### 3.5. Trans-Stadial Transmission of GRBV by S. festinus Adults Derived from Instars That Emerged on Infected Free-Living Vines

The occurrence of trans-stadial transmission of GRBV from Vcal hybrid vines was assessed by individually transferring nymphs of *S. festinus* that were born on infected Vcal hybrid vines to detached snap bean trifoliates (Figure 2). After 3–3.5 weeks (second instars), 2.5–3 weeks (third instars), and 2–2.5 weeks (fourth instars), respectively, adult *S. festinus* were collected immediately following emergence and tested for GRBV by qPCR. Of the adults, 20% (3 of 15) of second, 38% (5 of 13) of third, and 54% (7 of 13) of fourth instar-derived specimens tested positive for GRBV by qPCR. In addition, no molted skins (exuviae) shed upon adulthood on snap bean trifoliates tested positive for GRBV (0%, 0 of 9), regardless of the nymphal development stage. As expected, none of the adults (0%, 0 of 5) derived from instars from the colony maintained on healthy snap bean plants that were transferred to detached snap bean trifoliates tested positive for GRBV in qPCR. These results were consistent with GRBV persistence through the molt of *S. festinus* nymphs that acquired the virus from infected Vcal hybrid vines.

### 3.6. GRBV Titer in S. festinus Adults Derived from Instars That Emerged on Infected Free-Living Vines

**Figure 5:**
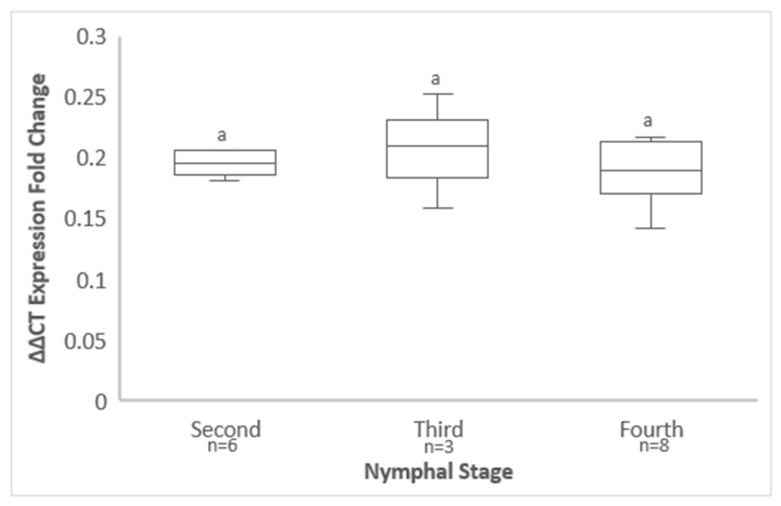
Quantification of grapevine red blotch virus (GRBV) by qPCR in *Spissistilus festinus* nymphal progeny born on GRBV-infected free-living vines, transferred to healthy, detached snap bean trifoliates as second, third, or fourth instars and tested upon emergence as adults. Error bars indicate standard error of the mean. Lowercase lettering indicating insignificant differences between ΔΔCt expression fold change values (2^(−ΔΔCt)^), as determined by Tukey’s honestly significant difference test (*p* < 0.05), are shown above standard error bars.

An increase in GRBV titer in adults derived from the third compared with the second instars that emerged on Vcal hybrid vines was observed, as demonstrated by a decrease in mean ΔΔCt values over time, but a decrease in GRBV titer was observed in adults resulting from fourth instars that emerged on Vcal hybrids, as shown by an increase in mean ΔΔCt expression fold change values (Figure 5). However, no statistically significant differences between 2^(−ΔΔCt)^ values were obtained between adults derived from second and fourth instars (*p* = 0.633), second and third instars (*p* = 0.979), or third and fourth instars (*p* = 0.623) (Figure 5). These results revealed that virus titer among *S. festinus* adults derived from nymphs that acquired GRBV from infected Vcal hybrid vines was independent of the nymphal developmental stage.

### 3.7. Transmission of GRBV by S. festinus Adults Derived from Instars That Emerged on Infected Free-Living Vines

Petioles of excised snap bean trifoliates were collected and tested for GRBV by qPCR at the onset and conclusion of the trans-stadial transmission assays. At the onset of the assays, none of the petioles tested positive for GRBV (0%, 0 of 12). Upon conclusion of the assays, all the petioles exposed to fourth instars and resultant adults (100%, 3 of 3) tested positive for GRBV and most of the petioles exposed to third (66%, 2 of 3) and second (66%, 2 of 3) instars and resultant adults tested positive for GRBV. As expected, none of the snap bean petioles on which colony-reared *S. festinus* fed were positive for GRBV (0%, 0 of 3). No difference in virus titer was found among snap bean trifoliates that were introduced to second (2^(−ΔΔCt)^ = 0.401), third (2^(−ΔΔCt)^ = 0.399), or fourth (2^(−ΔΔCt)^ = 0.404) instars (*p* = 0.533). These results documented transmission of GRBV from Vcal hybrid vines to detached snap bean leaves and demonstrated the vectoring capacity of *S. festinus* adults derived from nymphs who primarily acquired the virus on infected Vcal hybrid vines, although we cannot entirely exclude the possibility that the virus may have been acquired between molts from snap bean tissue that may have become infected following inoculation by viruliferous nymphs.

## 4. Discussion

In this study, we investigated the ability of *S. festinus* to transmit GRBV within and between two *Vitis* hosts: free-living Vcal hybrid vines and cultivated Vvin Cf grapevines. Transmission assays validated our hypothesis on the role of *S. festinus* in transmitting GRBV from infected Vcal hybrid vines to healthy Vcal hybrid vines and from infected Vcal hybrid vines to healthy Vvin Cf vines. Transmission also occurred from infected Vvin Cf vines to healthy Vcal hybrid vines. Together, these results suggest a role of infected free-living vines in red blotch disease epidemiology.

Grapevine viruses are increasingly identified in free-living vines, including Vcal and its hybrids [26,39,40] [this study], *V. riparia* [29], *V. sylvestris* [41], *V. labruscana* [42], *V. rupestris* [43], *V. cinerea* [35], *Vitis coignetiae* [44], and unidentified species [43], as well as the related *Ampelopsis cordata* [45]. Interestingly, among the different viruses identified in free-living vines, GRBV [this study] and grapevine vein clearing virus [45] are the only two viruses known to be transmitted from and to free-living vines.

Although GRBV was present in infected Vcal hybrid vines grown in the greenhouse, the virus was unevenly distributed in the leaves but present throughout the vines. This was unexpected and based on a marked preferential distribution of GRBV in basal (i.e., older) leaves of infected Vvin cultivars in the greenhouse and vineyard [37]. Nonetheless, understanding the viral distribution in shoots of Vcal hybrid vines allowed for a more targeted approach to virus acquisition by *S. festinus*. As a result, a higher rate of transmission was obtained when *S. festinus* fed on the middle shoots, despite no differences in virus titer throughout the Vcal hybrid vine shoots.

Varied rates of transmission were observed depending on the donor and recipient tissue type with a positive association between Vcal hybrid tissues and transmission efficiency of GRBV by *S. festinus* (Table 2). This result could be explained by a variance in tissue tropism among the two *Vitis* hosts. Indeed, many geminiviruses exhibit differences in tissue tropism patterns depending on virus-encoded factors and plant protein interactions [46]. Plant protein interactions have also been demonstrated to facilitate higher rates of acquisition by insect vectors of luteoviruses [47]. The localization of GRBV in plant host tissues and the identification of protein interactions facilitating the passage of GRBV across tissue barriers in *Vitis* hosts and *S. festinus* could provide clarity for this hypothesis. Beyond tissue tropism, an alternative explanation for unequal transmission rates is that *S. festinus* feeding behavior may differ between the two *Vitis* hosts, possibly resulting in greater rates of acquisition and GRBV transmission from Vcal hybrid vines. Indeed, behavioral differences were observed on Vcal hybrid vines in comparison to Vvin Cf vines. The fact that Vvin is not a primary feeding and reproductive host of *S. festinus* [48] was confirmed in this study. Adult *S. festinus* tend to act as a solitary species on Vvin Cf, spatially dispersing throughout a vine canopy and only aggregating while mating. On Vcal hybrid vines, however, we observed an increase in gregariousness among *S. festinus* adults, with insects on caged Vcal hybrid vines tending to aggregate at the tops of shoots. Host-specific behaviors by *S. festinus* have also been documented on Vvin Cf vines as compared to snap bean plants, as demonstrated by differences in the AAP, IAP, and transmission rates of GRBV on these two hosts [31]. More work is needed to determine how the tissue tropism and variation of *S. festinus* social and feeding behaviors on distinct plant species affect GRBV acquisition and transmission.

The trans-stadial transmission of GRBV by *S. festinus* nymphs was documented with Vcal hybrid vines [this study] and cultivated vines [31]. In previous experiments, the time of exposure to GRBV-infected Vvin Cf vines by first, second, and third instars correlated with GRBV titer in the insects as adults [31]. This trend was explained by increased feeding time and, thus, an increased capacity to acquire the virus for third instars, relative to second and first instars. Our data on Vcal hybrid vines included a fourth instar stage, which has not previously been recorded from reproduction on a *Vitis* spp. Interestingly, we observed a slight but statistically insignificant reduction in GRBV titer in their resultant adults. This result may be explained by variations in selective feeding behavior of *S. festinus* based on developmental stage, as previously documented on soybean [49]. First and second instars of *S. festinus* do not girdle soybean stems, but rather pierce the host plant by random feeding punctures. Interestingly, third instars cause stem girdling on upper petioles and main stems [49], while fourth instars are the most injurious and cause stem girdling primarily at the bases of stems [49]. If similar feeding and tissue preferences are expressed on *Vitis* spp., behavioral differences associated with distinct *S. festinus* development stages could explain the increased virus titer in adult insects which were transferred from infected vines at later nymphal stages [31], and an alteration in feeding behavior from probing (second and third instars) to girdling (fourth instars) could explain the reduction in overall GRBV titer we observed in adults that were moved from infected Vcal hybrid vines to snap bean trifoliates as fourth instars.

There is a striking difference between the proportion of viruliferous adult insects derived from nymphs that emerged on infected Vcal hybrid vines (37%, 15 of 41) reported in this study and the proportion previously reported on infected Vvin Cf (100%, 21 of 21) [31]. This difference is most likely explained by the higher overall virus titer of donor plant material from cultivated vines in comparison with material from Vcal hybrid vines. Furthermore, behavioral differences between nymphs on the two *Vitis* host species could explain the difference in the abundance of viruliferous *S. festinus* adults following trans-stadial transmission. Indeed, nymphs on Vcal hybrid vines were more mobile and were found more frequently on the undersides of newly emerging leaves, whereas those on Vvin Cf vines were more sedentary and primarily located on shoots and petioles. As a result, nymphs on Vcal hybrid vines may have had fewer opportunities to feed and acquire GRBV and thus to transmit the virus through molt. More work on insect behavior with regards to virus acquisition by different nymphal development stages is needed to address these different hypotheses.

The trans-stadial transmission of GRBV may have implications for red blotch disease management if *S. festinus* oviposition occurs on GRBV-infected grapevines in commercial vineyards or on GRBV-infected free-living vines. Favorable conditions for nymph development could result in viruliferous adults capable of transmitting the virus following acquisition without ever feeding on an infected vine. This method of virus acquisition by the insects may be a possible new transmission route of the virus. Such possibility should be investigated in vineyard ecosystems, for instance in northern California and southern Oregon, to inform whether populations of *S. festinus* should be targeted as a component of red blotch disease management.

*Spissistilus festinus* are most abundant in commercial vineyards in Napa Valley during June and July, but their presence has been reported beyond the summer months [31,32]. Some plant species used as cover crops in production vineyards are preferred feeding or reproductive hosts of *S. festinus*, but Vvin is not one of them [48]. However, cover crops are commonly tilled or mowed during late spring in northern California [50]. Thus, it seems that the full panel of plant species that could serve as feeding and reproductive hosts of *S. festinus* have not yet been identified in vineyard ecosystems. This could be an interesting research topic to further advance our limited understanding of red blotch disease epidemiology.

*Spissistilus festinus* is not an efficient vector of GRBV as transmission occurs at a low rate from infected Vvin to healthy Vvin [33,51] [this study]. In a previous study, the directional spread of GRBV was predicted to occur predominantly from vineyards hosting GRBV inoculum to free-living vines in riparian habitats [26]. This prediction, however, did not discount the potential of free-living vines to act as a source of virus inoculum, especially since *S. festinus* are known to visit free-living vines in riparian areas [31]. Our results suggest that studies to test whether the removal of free-living vines in proximity to vineyards has an impact on virus spread into and within a vineyard should be considered a high priority for growers in northern California, where the secondary vector-borne spread of GRBV has been confirmed [3,27,28,29,48]. The same recommendation may also apply to southern Oregon, where *V. riparia* has been found infected with GRBV [29].

## Figures and Tables

**Table 1:** Distribution of grapevine red blotch virus (GRBV) in *Vitis californica* hybrid vines.

Leaves ^a^						
Free-Living Vine	Top A	Top B	Middle A	Middle B	Bottom A	Bottom B
Infected 1	2/3	2/3	2/3	2/3	2/3	1/3
Infected 2	1/3	2/3	1/3	0/3	1/3	2/3
Total ^b^	3/6	4/6	3/6	2/6	3/6	3/6
	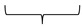		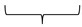		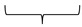	
Grand total ^c^	7/12		5/12		6/12	
Healthy	0/3	0/3	0/3	0/3	0/3	0/3

^a^ Two free-living vines (1 and 2) growing in the greenhouse were selected for this analysis. Three leaf samples from two shoots (A and B) and three shoot locations (bottom, middle, and top) of each vine were collected and tested for GRBV by qPCR. Data represent the number of leaf samples that tested positive for GRBV in qPCR over the total number of leaf samples tested. The free-living vines were first generation hybrids of *V. californica* and *V. vinifera* ‘Sauvignon blanc’; ^b^ Data represent the total number of leaf samples from distinct shoots and shoot locations that tested positive for GRBV in qPCR over the total number of leaf samples tested; ^c^ Data represent the total number of leaf samples from distinct shoot locations that tested positive for GRBV in qPCR over the total number of leaf samples tested.

**Table 2:** Transmission of grapevine red blotch virus (GRBV) by *Spissistilus festinus* from and to *Vitis californica* hybrid vines and to and from ‘Cabernet franc’ vines.

Virus Recipient Tissue			
Virus Donor Vine	Cultivated ^a^	Free-Living ^b^	Total ^c^
Cultivated	5/27 ^c^ (19%)	3/3 (100%)	8/30 (27%)
Free-living	2/13 (15%)	10/13 (77%)	12/26 (46%)
Total	7/40 (18%)	13/16 (81%)	20/56 (36%)

^a^ Cultivated grapevines were *Vitis vinifera* ‘Cabernet franc’; ^b^ Free-living vines were first generation hybrids of *V. californica* and *V. vinifera* ‘Sauvignon blanc’; ^c^ Data represent the total number of excised leaves that tested positive for GRBV in PCR over the total number of excised leaves exposed to *S. festinus* specimens.

## Data Availability

Raw data will be made available upon request.
